# A Structure-Based Analysis of the Evolution of Transcription Factors of the FNR/CRP Family

**DOI:** 10.3390/biom16020189

**Published:** 2026-01-26

**Authors:** Juan C. Fontecilla-Camps

**Affiliations:** Metalloproteins Unit, Université Grenoble Alpes, CEA, CNRS, IBS, F-38000 Grenoble, France; juan.fontecilla@ibs.fr

**Keywords:** transcription factor, protein synthesis, allostery, FNR, CRP, cAMP: X-ray structure, salt bridge, phosphate binding cassette

## Abstract

The X-ray structural analysis of the N-terminal domain cavity from eleven transcription regulators (TFs) of the Fumarate Nitrate Reduction regulator/cAMP Regulator Protein family shows several significant trends. The conservancy of effector-binding phosphate binding cassette features in three TFs suggests a closer connection among them than the one obtained through the comparison of overall amino acid sequences. Conversely, there are also three clearly different allosteric activation mechanisms, which most likely evolved independently. Interestingly, several TFs of this family adopt the DNA-binding conformation without binding any ligand; instead, the buried region corresponding to the “allosteric” cavity is partially filled with salt bridges (which is also the case for two allosteric apo TFs). One plausible conclusion from these observations is that the non-allosteric TFs evolved from an allosteric counterpart and used salt bridges to fill and stabilize the formally polar ligand-binding cavity. O_2_-sensing TFs share some residues in the relevant N-terminal domain cavity and might have had an already non-allosteric common ancestor.

## 1. Introduction

The regulation of gene transcription is one of the most fundamental biological processes, which is essential for cell growth, reproduction, and adaptation. One essential aspect of early life evolution was the need to preserve the genetic material from destruction by environmental causes. For instance, the enzymatic elimination of the 2′C-OH group in the ribose sugar, a component of RNA, to generate deoxyribose for DNA, removed problems related to the hydrolysis of the former, but it also restricted DNA to be an informational macromolecule.

Although DNA is less susceptible to undergoing chemical attack than RNA, it remains a large macromolecule that needs to be protected. This problem certainly worsened as the complexity of the (proto-)organisms increased. For instance, the DNA of the contemporary unicellular Gram-negative prokaryote *Escherichia coli* (*Ec*) contains about 5.0 × 10^6^ nucleotides corresponding to over 4000 genes [1]. This circular chromosomal DNA not only has to be preserved, but it also needs to be compacted over 1000-fold to be fitted inside the *E. coli* cell [2]. The necessary compaction of DNA, generally called supercoiling, has been proposed to be mediated by small basic proteins called Nucleoid-Associated Proteins (NAPs). Although NAPs are known to bind DNA with low-sequence specificity, it has become apparent that they also regulate transcription [3]. This would be a case of exaptation [4,5] where some proteins, which initially bound DNA to supercoil it, later became regulating transcription factors (TFs). In fact, the formal distinction between TF and NAP has become blurred, and these two protein classes are thought to form a continuous series going from highly specific ones, acting on a few genes, to those that have a pervasive role in transcription [6].

## 2. Background

**Figure 1:**
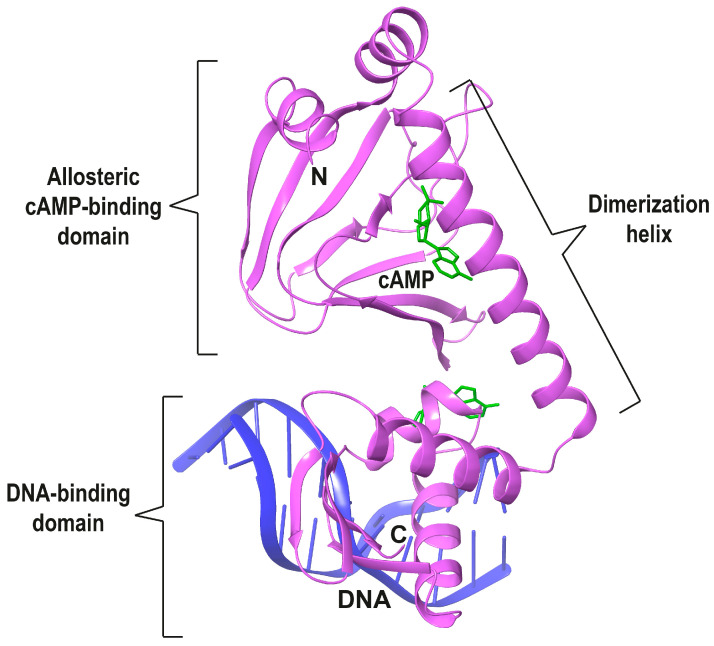
Ribbon depiction of one monomer from the dimeric *Ec*CRP (purple) with allosteric cAMP bound (green) and complexed to DNA (blue), pdb code 2CGP, [8]. A second cAMP molecule bound between the cAMP-binding and DNA-binding domains is also found in the structure. See below for depictions of the dimeric *Ec*CRP structure.

One well-studied group of TFs is the Fumarate Nitrate Reductase regulator (FNR)/cyclic AMP Receptor Protein (CRP) family. FNR/CRP TFs are homodimers, whose monomers are composed of a versatile N-terminal domain (N-terD) and a C-terminal DNA-binding helix-turn-helix domain (DBD), which are connected by a long dimerization C-helix. The first crystal structure of cAMP-bound *Ec*CRP, one of the most studied TFs of this family, was published in 1981 [7]. The more recent structure of an *Ec*CRP-DNA complex [8] is shown in Figure 1. The two types of domains of this family are found independently associated in proteins displaying a vast array of different functions. For instance, the allosteric phosphorylating protein kinase A undergoes significant conformational changes upon cAMP binding [9]. The structural comparison of the cyclic nucleotide-binding (CNB) domains of this kinase with a cyclic nucleotide-gated channel indicates that they share a C-helical region (Figure 3d in [9]) equivalent to the dimerization C-helix of FNR/CRPs (Figure 1).

Interestingly, in tetrameric protein kinases, the two homologous CNB domains of each of the two regulatory subunits are linked by their C-helical stretches; however, these subunits do not form a homodimer connected by C-helices, as in FNR/CRPs [10]. This observation suggests that the fusion of the allosteric cAMP-binding and DNA-binding domains of FNR/CRP TFs and their dimerization took place after the evolution of cAMP-dependent enzymatic activities, such as the regulated phosphorylation by kinases in metabolic central processes.

Signal transduction within the FNR/CRP family is defined as a “one-component” system, a configuration that is abundant in prokaryotic TFs [11]. These systems lack histidine kinase and response regulator domains, which are typical of more complex “two-component” TFs [12]. This observation suggests that phosphorylation was not part of transcription regulation in very early metabolisms. One-component systems are considered to be ancestral to two-component systems, and it is possible that the former were the only class of signal transducers present in the last universal common ancestor (LUCA) [11].

Although DBDs, like the ones found in FNR/CRPs, are present in Archaea and Bacteria, their association with a ligand-binding N-terD is only found in the latter [13]. A very recent study by Krishnaswamy et al. has reported sequence and phylogenetic analyses concerning the evolution of the DBDs in the FNR/CRP family [13]. Besides binding to DNA, the DBD activates class 1 transcription by binding to the C-terminal domain of RNA polymerase. The authors proposed that the ancestral DBDs were more similar to those found in a diverse class of “CRP-like” proteins distinct from the well-studied CRP and FNR TFs found in *E. coli* [13]. These conclusions do not agree with those reported earlier by Matsui et al., who, using a computer-based genomic “spectral clustering” analysis, postulated that an ancestral FNR protein, involved in nitrogen fixation, originated the contemporary FNR/CRP family [14].

The conflicting conclusions of these two studies reflect the inherent complications associated with building reliable prokaryotic phylogenetic trees; the problem is thought to be caused by both extensive horizontal gene transfer (HGT) and inaccuracies due to differences in evolutionary rates between lineages [15].

In general, protein foldings are more conserved than amino acid sequences [16], and this is clearly the case for the FNR/CRP family. Consequently, it will make sense to compare the TFs of this family with available 3D X-ray structures to try and shed light on their evolution. Because of the inherently complex evolutionary pressure undergone by these regulatory proteins, our analyses will be restricted to the cavity buried in the N-terD -which in *Ec*CRP binds the allosteric cAMP ligand (Figure 1). The cAMP-binding region of the *Ec*CRP structure will be used as a reference when comparing the corresponding cavities of the other TFs.

## 3. Structural Similarities and Differences in the Buried Cavity of the N-Terminal Domain

Our working hypothesis is that structural similarities in the N-terD “allosteric” cavity found among FNR/CRP TFs will help establish their evolutionary relationships from a different perspective than the comparison of overall amino acid sequences.

### 3.1. The Allosteric Cavity in Members of the FNR/CRP Family

**Figure 2:**
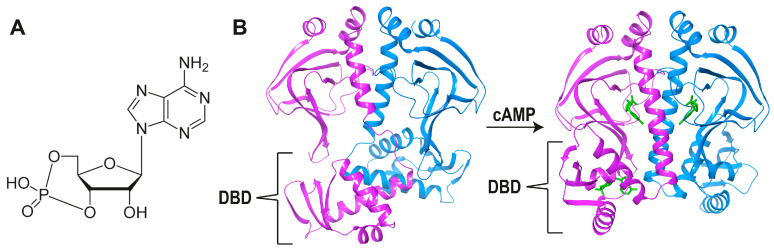
(**A**). The structure of cyclic AMP. (**B**). Ribbon representation of the conformational change in dimeric *Ec*CRP upon cAMP binding (pink and blue monomers, pdb codes apo 4N9H [19] and holo 4HZF [20]). DNA-binding domains (DBDs) are indicated; cAMP molecules are depicted with green sticks.

*Ec*CRP. Although cAMP-bound *Ec*CRP is involved in many aspects of carbon metabolism through allosteric transcription regulation, it also displays DNA supercoiling activity [17]. Because apo-CRP can have an NAP-like global effect on gene expression, it has been suggested that this protein represents an intermediate stage in the evolution of a ligand-dependent allosteric TF from a NAP [17,18]. However, the apo form of *Ec*CRP has its DBDs oriented very differently when compared to the holo form (Figure 2B). In addition, apo-*Ec*CRP binds to DNA with either low or no specificity.

The cAMP-dependent *Ec*CRP allosteric activation mechanism has been proposed to be based on the H-bonds formed by the N^6^ group of the bound nucleotide with (*cis*) Thr128 from one C-helix and (*trans*) Ser129’ from the other [21]. These H-bonds bring the two C-helices closer together and closer to cAMP. This reorientation allows several hydrophobic residues on both helices to interact, which extends their length by 1.5 turns. These changes also modify the helical content and orientation of the DBDs, placing them in the right configuration for cognate DNA binding (Figure 2B) [21]. The most conserved feature of the cAMP-binding cavity has been called the “phosphate binding cassette” (PBC) [9], which, as we will see below, shares some structural elements with other TFs of the family. A second cAMP site is present in one of the monomers of the holo *Ec*CRP (Figure 1 and Figure 2B), but it does not seem to play an in vivo allosteric role.

NtcA. Another example of allosteric regulation by a TF from the FNR/CRP family is the cyanobacterial nitrogen regulator NtcA [22]. The crystal structures of apo NtcA and its complex with its 2-oxoglutarate (2-OG) effector have been reported [23,24]. 2-OG is a metabolite of the Krebs cycle, a hub of cell metabolism of generally accepted great antiquity.

When nitrogen becomes scarce, the reductive amination of 2-OG to generate glutamate is impaired, and 2-OG accumulates; under these conditions, NtcA binds this metabolite and positively regulates nitrogen assimilation [22]. The conformational transition between the apo and complexed states is much simpler in NtcA than in *Ec*CRP (Figure 2); it mostly involves a relatively minor change in the orientation of the dimerization C-helices. This change is mainly caused by 2-OG binding to (*cis*) Arg129 from one C-helix and (*trans*) Glu134’ from the other.

**Figure 3:**
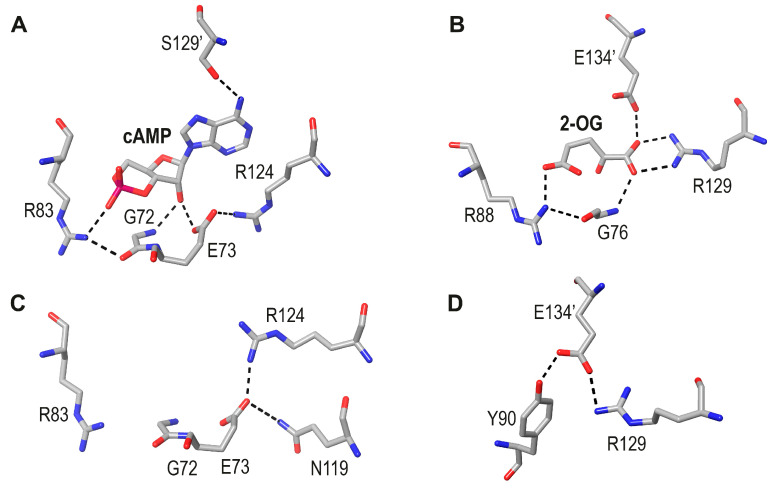
Depiction of TF ligand-binding regions in holo and apo *Ec*CRP and NtcA: (**A**). *Ec*CRP + cAMP (pdb code 4HZF; [20]). (**B**). NtcA + 2-OG (pdb code 3LA2, [24]). (**C**). Apo *Ec*CRP (pdb code 4N9H, [19]. (**D**). Apo NtcA (pdb code 3LA7, [24]).

Although NtcA is the only known member of the FNR/CRP family to be co-activated by another protein, its complex with 2-OG is similar to the cAMP-*Ec*CRP complex [24] (Figure 3A,B). Thus, one of the O atoms of the 5-COO^−^ group of 2-OG and one of the O atoms of the phosphate group of cAMP show equivalent interactions with Arg88 and Arg83. These residues are H-bonded to the respective mainchain O atoms of Gly 76 and Gly 72. In addition, the mainchain N-H groups of these Gly residues interact with the corresponding ligands. Interestingly, and as already noted by Zhao et al. [24], the helix-stabilizing *Ec*CRP (*trans*) Ser129’-cAMP bond is stereochemically equivalent to the NtcA (*trans*) Glu134’-2-OG bond. Thus, similar intermonomer interactions are found in the two TFs, although in NtcA, the longer side chain of a Glu residue is required to reach the smaller 2-OG.

In *Ec*CRP these motifs have been defined as part of the conserved PBC mentioned above [9]. Arg129 of NtcA, which forms a bidentate salt bridge with 2-OG, has a corresponding Arg124 in *Ec*CRP; however, this residue makes a salt bridge interaction with Glu73 in a different orientation due to the presence of the cAMP adenine base (Figure 3A). Interestingly, for the discussion below, in apo *Ec*CRP, this salt bridge is conserved, but its orientation is very different (Figure 3C). Also, when holo and apo NtcA are compared, a salt bridge between R129 and (*trans*) Glu134’ is observed in the latter (Figure 3D).

Intriguingly, and based on amino acid sequence analyses, CRP and NtcA have been assigned to clearly different branches of the FNR/CRP superfamily (Figure 2 in [25]). However, unless one invokes a strong convergent evolutionary process, the observed similarities in their cavities are likely to represent conserved features already found in a common allosteric ancestor. The next point to address is the possible relationship between that ancestor and the two contemporary effectors. cAMP is involved in many types of stress responses [26], so it seems to be a more versatile metabolic effector than 2-OG. Conversely, 2-OG is a precursor of the essential proteogenic Glu and Gln amino acids. In a primordial proto-cell amino acid synthesis might have been more essential than allostery.

Concerning the allosteric mechanism elicited by each of these ligands, *Ec*CRP activation involves major conformational changes (Figure 2B), whereas this is not the case for NtcA [24]. From a parsimonious viewpoint, it might then be proposed that NtcA should be closer to the common ancestor than *Ec*CRP. What seems clear is that the evolutionary paths of the two proteins were directly connected.

CprK. Several bacterial species are able to conserve energy through halorespiration [27]. In the strictly anaerobic, Gram-positive *Desulfitobacterium* spp. [28], the expression of halorespiratory genes is controlled by the allosteric CprK TF, which binds the *o*-chlorophenolacetic acid (OCPA) effector [29]. Several crystal structures of CprK have been reported with and without its ligand, and hypotheses about the stereochemistry of the allosteric process have been proposed [30,31,32]. The halogenation of *o*-phenolacetate to yield OCPA is essential for its recognition by this TF and its activation [29]. The general conclusion is that halogenation favors the deprotonation of the *o*-phenolacetate -OH group, which is needed for the initially weak OCPA binding to one of the CprK monomers.

**Figure 4:**
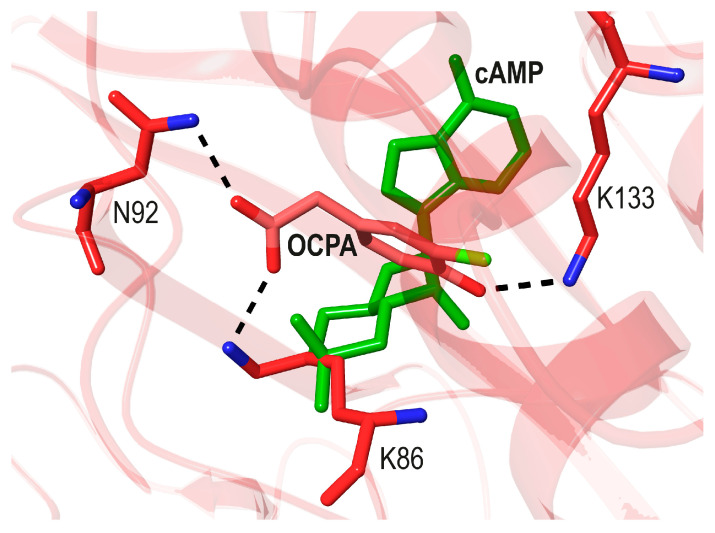
Superposition of OCPA bound to CprK (red C atoms, green Cl atom, and pink ribbon mainchain tracing, pdb code 3E5U, [30]) with cAMP bound to *Ec*CRP (green cAMP, pdb code 4HZF, [20]). The interactions of OCPA with Asn92, Lys86, and Lys133 are highlighted.

The crystal structure of the CprK-OCPA complex shows that the deprotonated phenoxide ion forms a salt bridge with the -NH_3_^+^ group of Lys133 from the *cis* C-helix (Figure 4). OCPA binding introduces several major conformational changes that greatly increase the affinity of the other CprK monomer for this ligand [30,32]. After the binding of the second OCPA, the DBDs of the holo CprK dimer are well oriented to bind its cognate DNA regions. This allosteric process has been defined as a case of “extreme positive cooperativity” [30]. Although OCPA binds to a site in CprK topologically equivalent to the sites of *Ec*CRP for cAMP and NtcA for 2-OG, the residues involved in its binding are very different (compare Figure 3 to Figure 4). It should also be mentioned that when CprK is in vitro exposed to O_2_, it forms an interdomain Cys11-Cys200’ disulfide bridge. The oxidized TF can still bind OCPA but is unable to recognize its cognate DNA. However, results obtained using the corresponding Cys → Ser mutants did not support a physiological role for the disulfide bridge in CprK expressed in aerobically grown *E. coli* cells [33].

In summary, CprK may be a distinct, more recent addition to the FNR/CRP family. In fact, the comprehensive amino acid sequence analysis mentioned above has placed CprKs as a separate branch of this family [25]. Several other observations agree with this notion. Firstly, phylogenetic and structural analyses suggest that there might be evolutionary and functional relationships between the six known families of halogenating enzymes and α/β hydrolases, acid phosphatases, peroxidases, chemotaxis phosphatases, oxidoreductases, and the SAM hydroxide adenosyltransferases, respectively [34]. This suggests that each of the halogenating proteins has evolved from a pre-existing enzyme with similar catalytic properties. Secondly, halogen-containing compounds, such as OCPA, are mostly the polluting result of past and present industrial and agricultural human activities [30]. For this reason, it is not possible to rule out the relatively recent evolution of some of the halogenating enzymes in halogen-metabolizing bacteria to conserve energy in a respiration-type metabolism. Thirdly, the extreme cooperativity of the CprK allosteric mechanism is not found in other FNR/CRP family members [30].

### 3.2. Negative Allostery in the FNR/CRP Family

As mentioned above [13] and further discussed below, many of the functional and structural conclusions based on *Ec*CRP, and by extension on NtcA, do not apply to all the members of the FNR/CRP family. Thus, *effector binding can also have a negative effect on transcription regulation*.

**Figure 5:**
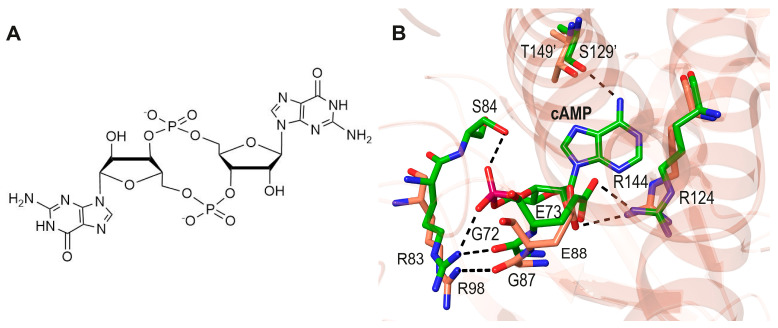
(**A**). bis-(3′–5′)-cyclic di-guanosine monophosphate. (**B**). Superposition of holo *Ec*CRP + cAMP (green C atoms, pdb code 4HZF, [20]) and apo *Xc*Clp (beige carbon atoms and mainchain ribbons, pdb code 3IWZ, [35]).

Clp. The Clp TF from the plant pathogen *Xanthomonas campestris* (*Xc*) binds to cognate DNA without fixing any ligand [35]. However, the binding of the bacterial allosteric inhibitor bis-(3′–5′)-cyclic di-guanosine monophosphate (c-d-GMP, Figure 5A) to *Xc*Clp causes its dissociation from DNA. The crystal structure of the apo form of this TF, as determined by Chin et al., shows that it adopts an intrinsically active conformation for DNA binding [35]. These authors have also explored possible c-d-GMP binding sites in *Xc*Clp by molecular docking using a home-made program. Their preferred model had c-d-GMP bound between the TF N-terD and DBDs, close to the C-helix [35].

The negative allosteric mechanism displayed by *Xc*Clp [35,36] is the most unusual one reported so far for a member of the FNR/CRP family. Examination of the N-terD cavity shows that the orientation of the PBC Arg83-Gly72 and the Arg88-Gly76 motifs, involved in the respective binding of the acidic functions of cAMP and 2-OG (Figure 3), is also found in *Xc*Clp (Arg98-Gly87 motif, Figure 5B). In addition, the *Xc*Clp salt bridge between Glu88 and Arg144 is similar to the one found in *Ec*CRP between Glu73 and Arg124 (Figure 5B). Furthermore, although cAMP is not present in *Xanthomonas* [37], it competed with c-d-GMP for binding to the related Clp from *X. anoxopodis* when added at mM concentrations [36,37]. This observation raises the possibility that the c-d-GMP effector, which has two phosphate groups chemically equivalent to the one found in cAMP (see Figure 2A and Figure 5A), could bind close to the Arg98-Gly87 motif.

Because of its bulk, c-d-GMP binding to the N-terD cavity would result in major conformational changes in Clp, plausibly causing its dissociation from DNA. Such changes would also complicate an in silico prediction of the c-d-GMP docking process. If this notion is correct, the allosteric process of Clp would be almost the exact reverse of the one found in *Ec*CRP.

### 3.3. Different Allostery in Other Members of the FNR/CRP Family

PrfA. In the Gram-positive intracellular human pathogen *Listeria monocytogenes*, the expression of the most virulent genes is regulated by PrfA [38]. This TF is capable of binding to a consensus DNA sequence with low affinity in the absence of an effector [39]. However, its activity increases in an intracellular environment, and the crystal structure of the constitutively active G145S-PrfA variant showed the structuring of the helix-turn-helix motif of the DBDs by forming an H-bond between the Oγ of Ser145 and the O of Gly14 [39]. These observations strongly suggested that PrfA was an allosteric TF that would bind a specific effector. This hypothesis was confirmed when a glutathione synthase was shown to be essential for PrfA activity and reduced glutathione (GSH) increased it [40]. As reviewed in [41], glutathione is also known to play a central role in many other processes: redox buffering, response to oxidative stress, biosynthesis of iron–sulfur proteins, detoxification of metals and xenobiotics, redox signaling, apoptosis, and sulfur storage and transport. Its role in the remodeling of the transcriptional program of the pathogen involves a redox 2 GSH ⇔ GSSG + 2 H^+^ equilibrium. When *L. monocytogenes* is phagocytized by a macrophage, it is initially captured in a vacuole. There, both PrfA and GSH get oxidized (PrfA has four redox-active cysteine residues), and the oxidized TF can neither bind DNA nor be activated by GSSG. Next, the bacterium escapes from the oxidizing vacuole, and both PrfA and bacterial GSSG get reduced. In addition, *L. monocytogenes* can also get reduced GSH from the macrophage cytoplasm, and under these conditions, it colonizes the host cell [40].

**Figure 6:**
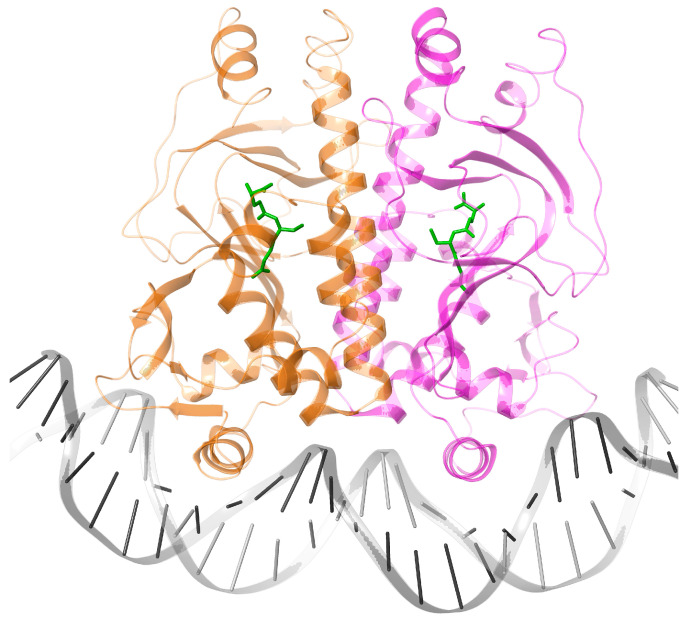
View of the DNA-GSH-PrfA crystal structure (PrfA, magenta and orange ribbons; GSH, green sticks; and DNA, gray ribbons and sticks; pdb code 5X6E [42]).

Besides the structure of the G145S-PrfA variant [39], several other crystal structures of PrfA are available, including complexes with DNA and GSH [42,43]. The effector binding causes conformational changes in the DBDs that are very similar to those found in the G145S-PrfA variant with or without bound GSH. The effector binds to a tunnel site between the N-terD and DBDs of the monomer (Figure 6). This ligand-binding region is clearly different from the cavity described for the effectors of *Ec*CRP, NtcA, and CprK (Figure 6). Indeed, a structural superposition of cAMP-*Ec*CRP and GSH-PrfA shows that the closest distance between atoms of the two ligands is about 4 Å. The dissimilar orientations of the effectors indicate that *Ec*CRP and PrfA have different allosteric mechanisms [43].

**Figure 7:**
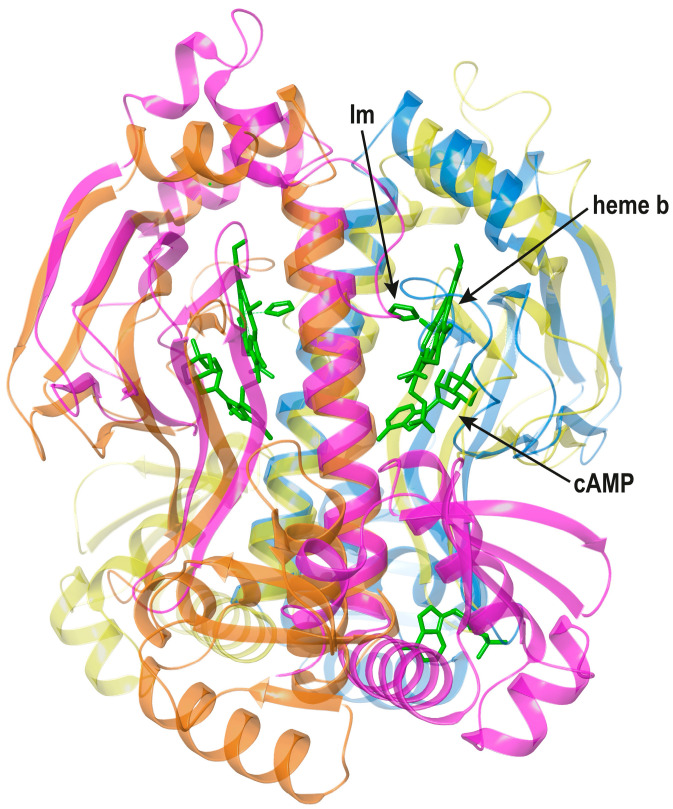
Superposition of *Ec*CRP (pdb code 4HZF, [20] blue and orange ribbons) and *Ch*CooA (pdb code 2FMY, [46] yellow and magenta ribbons, Im: imidazole). The cAMP and Im-bound heme b cofactors (green sticks) are labeled in the right-side monomer.

CooA. In bacteria such as *Rhodospirillium rubrum* (*Rr*), CO metabolism is controlled by the CooA TF that binds this gas to its heme b cofactor [44]. The first crystal structure of CooA showed that the Fe^2+^ ion bound to heme b was axially coordinated by His77 and, rather surprisingly, by the terminal amine nitrogen of Pro2 from the other monomer [45]. A later report of the related *Carboxydothermus hydrogenoformans* (*Ch*) CooA structure with heme-bound imidazole (Figure 7) indicated that, in vivo, CO should displace the *trans* Pro2 N-terminal ligand [46].

The functional properties of *Rr*CooA and *Ch*CooA, including different redox responses of their heme cofactors, such as the swap of an axial ligand, have been recently reviewed [47]. CooA coordinates heme b in its N-terD cavity (Figure 7). However, the cofactor is not located at the place of either cAMP in *Ec*CRP or GSH in PrfA. In fact, heme b binding requires the absence of a region corresponding to residues 72–82 in *Ec*CRP; furthermore, a longer N-terminal segment is needed for Pro2 to reach the heme of the other monomer [45].

Although, as shown, *trans* monomer ligand-binding effects are also found in *Ec*CRP and NtcA, they clearly differ from *trans* Pro2 binding to heme b in CooA. In the Im-bound *Ch*CooA crystal structure, the Pro2 -NH_2_ group is located 16 Å from the heme, whose plane rotates 30° about the normal to the porphyrin ring relative to the inactive *Rr*CooA structure [44]. In a new variant *Ch*CooA structure, which has one monomer in the active state, it was found that the N-terminal segment was located between the heme-binding and DB domains [48]. In this bridging position, the N-terminal segment helps orient the DBDs so that they adopt the DNA-binding configuration. This configuration has been defined as the “N-terminal Velcro model” [49]. In many respects this allosteric member of the FNR/CRP family is the one that displays the most elaborate conformational changes upon CO binding.

### 3.4. Non-Allosteric Members of the FNR/CRP Family

Some TFs bypass effector binding completely. We will first discuss three extremophile proteins that belong to this class.

SdrP/HB099/DdrI. SdrP and HB099 from *Thermus thermophilus* [50,51] and DdrI from *Deinococcus radiodurans* [52] are transcriptionally active without binding a ligand. The expression of sdrP mRNA increased in the stationary phase during cultivation at 70 °C. This increase was, to a great extent, a response to oxidative stress. The expression levels of the SdrP-regulated genes probably depend on the concentration of SdrP.

Although based on the sequences of their ribosomal RNAs and proteins, these two extremophile organisms have evolved from a common ancestor, extensive specific gene loss and acquisition have rendered them clearly different [15,53]. *T. thermophilus* is a Gram-negative thermophile that displays low resistance to ionizing radiation and desiccation, whereas *D. radiodurans* is a Gram-positive mesophile highly resistant to those two drastic environmental conditions.

**Figure 8:**
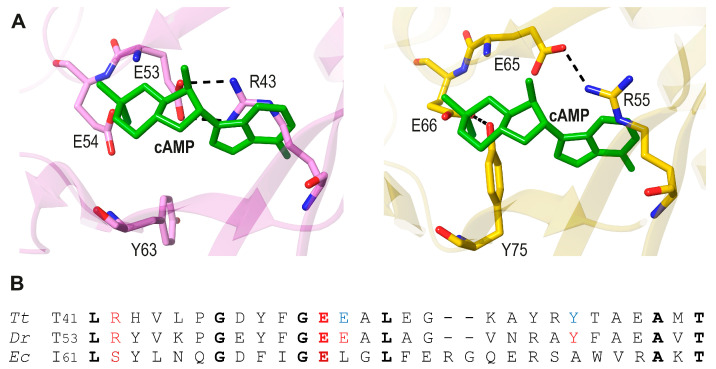
(**A**). Superposition of cAMP of holo *Ec*CRP (green, pdb code 4HZF [20]) to *T. thermophilus* HB099 (pink carbon atoms and mainchain ribbons (pdb code 3B02, [50] left), and to *D. radiodurans* DdrI (yellow carbon atoms and mainchain ribbons, pdb code 8YZ7, [52] right). Equivalent Arg43-Glu53 (left) and Arg55-Glu65 (right) salt bridges are highlighted. In addition, a H-bond between Glu66 and Tyr75 (light red in **B**) is observed in DdrI. The equivalent Glu54 and Tyr63 residues of HB099 (light blue in **B**) do not interact in the crystal structure. (**B**). Amino acid sequence alignment of the region shown in (**A**) for *T. thermophilus* (*Tt*), *D. radiodurans* (*Dr*), and *E. coli* (*Ec*). Residues discussed in the text are highlighted (bold: strictly conserved ones).

The HB099 and DdrI crystal structures show that the site corresponding to where the cAMP adenine base binds in *Ec*CRP is occupied by (*HB099*) Arg43-Glu53 and (*DdrI*) Arg55-Glu65 salt bridges (Figure 8A). These salt bridges help confer a “cAMP-bound-like” conformation to these TFs. Notably, the residue equivalent to Arg43 and Arg55 in *E. coli* is Ser53 (Figure 8B). 

Although it cannot be unambiguously determined, it seems structurally more parsimonious to conclude that cAMP-binding CRP predated the extremophile active apo-CRP versions because of its ubiquity [9]. After all, disabling the cAMP binding site would have required the stabilization of the observed salt bridges at the N-terD cavities, whereas optimizing cAMP binding to that site was probably a gradual, slow evolutionary process.

**Figure 9:**
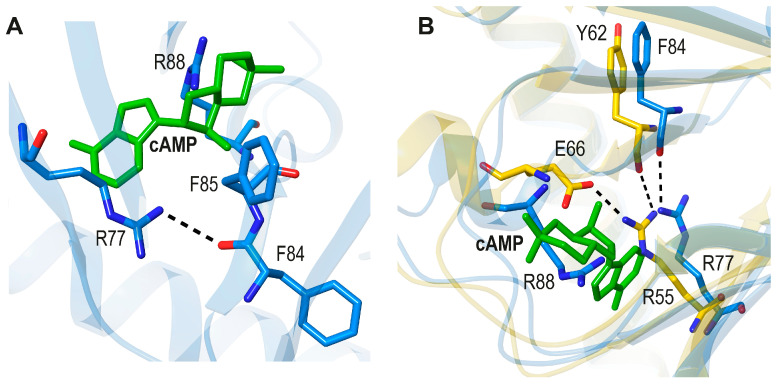
(**A**). Superposition of cAMP from holo *Ec*CRP (pdb 4HZF, [20] green) to *Pg*CRP (pdb code 2GAU, [57] blue carbon atoms and Cα chain ribbons). (**B**). Same as A plus *Dr*CRP (pdb code 8YZ7, [52] yellow carbon atoms and Cα chain ribbons).

PgCRP. Another effector-independent TF has been described in the Gram-negative and strictly anaerobic human oral pathogen *Porphyromonas gingivalis* (*Pg*) [54,55]. *Pg*CRP is involved in biofilm formation and adhesion to, and invasion of, oral keratinocytes. Like *Tt*SdrP and HB099 [50,51], *Pg*CRP activity is thought to be regulated by its concentration in the bacterial cell. Its crystal structure is also similar to the cAMP-*Ec*CRP complex structure [56]. A superposition of these two TFs shows that the side chains of Arg77 and Arg88 of *Pg*CRP occupy the flanks of the N-terD cavity (Figure 9A). They establish long-range interactions with main-chain carbonyl oxygens. Interestingly, the H-bond of Arg77 with the >C=O from Phe84 is structurally close to the one between Arg55 and the >C=O from Tyr62 in the *D. radiodurans* DdrI (Figure 9B).

### 3.5. Non-Allosteric O_2_-Sensing Members of the FNR/CRP Family

FNRs/FixK_2_. Three other TFs, which do not bind a ligand, are the O_2_-sensing [4Fe-4S]-FNRs from Gram-negative [58] and Gram-positive bacteria [59], and FixK_2_ from *Bradyrhizobium japonicum* [60].

**Figure 10:**
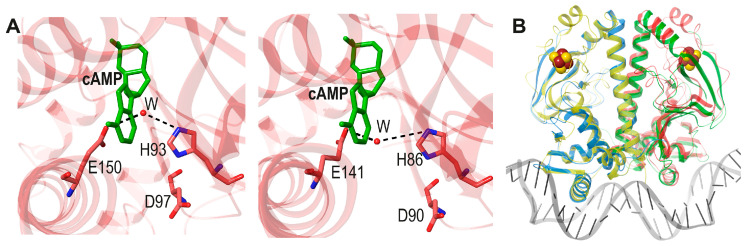
(**A**). N-terD cavities of *Af*FNR (left, pdb code 8QTO, [61]) and FixK_2_ (right, pdb code 4I2O, [60]). Respective residues His93, Asp97 and Glu150 and His86, Asp90 and Glu141 are labeled. The superposed cAMP from the cAMP-*Ec*CRP complex (pdb 4HZF, [20] green) is also shown as a reference. (**B**). View of the superposition of dimeric *Af*FNR (yellow and red mainchain ribbons, pdb code 8QTO) to the DNA-FixK_2_ complex (blue and green mainchain ribbons, gray DNA strands, pdb code 4I2O). [4Fe-4S] clusters are represented with red Fe and yellow S atoms.

The two types of iron–sulfur cluster-binding FNRs have very different sensing mechanisms. In FNRs from Gram-negative bacteria, a dimer → monomer transition, which results in loss of DNA binding, is caused by the disassembly upon O_2_ exposure of an N-terminally located [4Fe-4S] cluster (Figure 10B) [58]. Conversely, in the FNR from Gram-positive bacteria, the [4Fe-4S] cluster is located in the C-terminal DBD; this region is expected to be altered upon O_2_ exposure, which results in cluster decomposition, without causing a dimer–monomer transition [59].

FixK_2_ activates genes involved in nitrogen-fixation and in anoxic, microoxic, and endosymbiotic processes through an O_2_-sensing mechanism, which does not depend on an iron–sulfur cluster. FixK_2_ has a very redox-sensitive Cys-SH group (Cys183), which becomes oxidized by O_2_. Consequently, and in order to facilitate the manipulations, the crystallized species was the C183S variant [60].

DNA-bound dimeric FixK_2_ adopts a configuration that is very similar to the one displayed by the [4Fe-4S]-FNR from Gram-negative *Aliivibrio fischeri* (*Af*) [61] (Figure 10). Because the crystal structure of uncomplexed FixK_2_ is not available [60], it is not known whether it displays a similar dimeric conformation. What seems clear is that holo *Af*FNR should have a DNA-binding dimeric conformation in solution, as shown by the orientation of its DBDs in our crystal structure [61] (Figure 10B).

The *Af*FNR Cys-rich cluster-binding region has no visible counterpart in FixK_2_ (Figure 10B). In fact, both proteins display N-terminal disordered regions in their crystals (1–16 in *Af*FNR and 1–37 in FixK_2_) [60,61]. As expected, FixK_2_ lacks key residues that in FNR determine the dimer–monomer transition, and insuing loss of DNA binding, caused by O_2_ exposure and [4Fe-4S] cluster disassembly [61].

Although the overall amino acid identity between *Af*FNR and FixK_2_ is about 25%, three regions show significantly higher identities. As expected from their similar DNA binding sites, the respective helix-turn-helix DNA-binding sequences have 74% identity. Another region with a high identity score (69.2%) lines the cavity structurally equivalent to the allosteric one where cAMP binds in *Ec*CRP:

**Q**  **I**  T  **A**  **F**  **H_93_**  **L**  A  **G**  **D_97_**  L  V  **G** 

**Q**  **I**  G  **A**  **F**  **H_86_**  **L**  P  **G**  **D_90_**  V  F  **G** 

*Af*FNR and FixK_2_ have three homologous polar residues (FNR: His93, Asp97, and Glu150, and FixK_2_: His86, Asp90, and Glu141) (Figure 10A) and one conservative change (FNR Gln155 and FixK_2_ Glu146) in that cavity. This degree of conservancy of internal charged and polar residues is considerable; it strongly suggests that the common ancestor of FNR and FixK_2_ was already not allosteric.

These two TFs have very different O_2_-sensing mechanisms. The FixK_2_ redox-active Cys183 thiolate mentioned above is located at the DBD. There, it reacts with radical oxygen species (ROS) to form either sulfenic, sulfinic, or sulfonic acid derivatives, which hinder DNA binding [62]. This mechanism appears to be simpler than the dimer–monomer transition of Gram-negative FNR, suggesting that it may be older. *Af*FNR has two major insertions relative to FixK_2_: the three Cys [4Fe-4S] cluster binding loop (FNR res. 17–29) and the 180–190 loop. These two regions interact through van der Waals contacts and, upon cluster disassembly, the conformational change in the former loop would be transmitted to the DBD by the latter loop.

**Figure 11:**
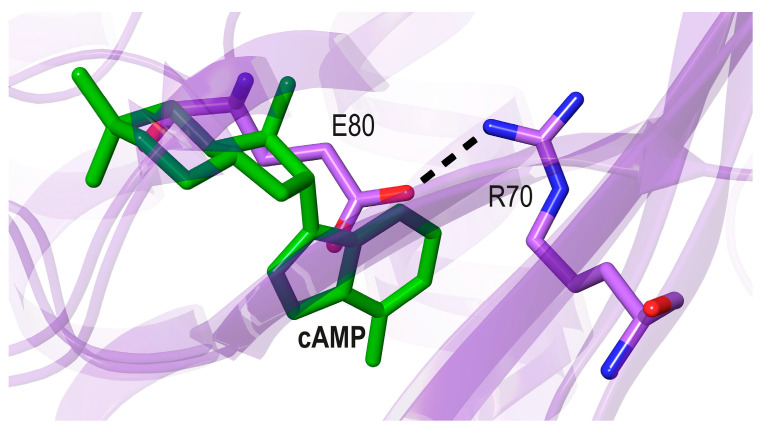
Superposition of the Cα chain of holo *Ec*CRP (pdb code 4HZF, [20]) + cAMP (green) to *Bacillus* sp. Gram-positive FNR (modeled AF052953.pdb, [63] purple carbon bonds and ribbons).

Although there is no X-ray structure available for an FNR from a Gram-positive bacterium, an Alphafold model of the FNR from *Bacillus subtilis* (*Bs*)—downloaded from the Uniprot website (AF052953.pdb) [63]—confirms the C-terminal position of the [4Fe-4S] cluster (not shown). Interestingly, the model also predicts the formation of a salt bridge between Arg70 and Glu80 in the N-terD cavity (Figure 11).

We have seen that FNR/CRP TFs have been subjected to intense evolutionary pressure to generate a large array of activities and mechanisms starting from a common, rather simple two-domain protein. The structures reviewed above show that when a key metabolic function (such as the regulation of gene expression) needs to be adapted to changing conditions Nature can display a vast array of solutions. A point worth mentioning is the possible antiquity of the connection between some FNR/CRP TFs and N_2_ fixation (NtcA, FixK_2_) and O_2_ metabolism (FNRs, FixK_2_), whose origins can be traced to 3.77 Ga [64] and 2.7–3.2 Ga [65], respectively. Both gases were already metabolized by ancestors of cyanobacteria.

## 4. Conclusions

A few major points deserve to be noted: 1. Conservancy of effector-binding PBC features of *Ec*CRP in NtcA and the PBC-like Arg-Gly motif in Clp, a case of negative allostery, suggests a closer connection among these TFs than the ones proposed based on overall amino acid sequences. 2. Although PrfA and CooA also use their N-terD for effector binding, their allosteric activation mechanisms are different from each other, and from the one mediated by cAMP in *Ec*CRP; it may be then concluded that in the FNR/CRP family, allostery independently evolved at least three times; 3. There are several non-allosteric TFs that do not bind any ligands; in SdrP, HB099, and DdrI, and the modeled *Bs*FNR, the “allosteric” cavity has been partially filled with buried salt bridges (also the case in apo *Ec*CRP and apo NtcA). These observations suggest that the non-allosteric TFs evolved from ligand-binding counterparts; not only would allostery have made more sense in metabolic regulation, but also filling in a pre-existing ligand-binding polar cavity with salt bridge-forming residues seems simpler than the opposite. 4. The less involved allosteric mechanism of NtcA relative to *Ec*CRP, and the metabolic role of 2-OG when compared to cAMP, suggest that 2-OG could have predated cAMP as an effector. 5. *Af*FNR and FixK_2_ have similar residues in the relevant N-terD cavity and could have had a common—already non-allosteric—ancestor.

The available TF structures display a functional progression from positive allosteric (activated by ligand) to negative allosteric (disactivated by ligand) to non-allosteric (no ligand). It is tempting to postulate that such progression could have an evolutionary sense.

## Data Availability

No new data were created or analyzed in this study. Data sharing is not applicable to this article.

## Abbreviations

The following abbreviations are used in this manuscript:

c-d-GMP: bis-(3′–5′)-cyclic di-guanosine monophosphate; CNB: cyclic nucleotide domain; DBD: DNA-binding domain, GSH: glutathione; N-terD: N-terminal domain; OCPA: *o*-chlorophenolacetic acid. TF: transcription factor; 2-OG: 2-oxoglutarate; PBC: phosphate binding cassette
